# Flavin adenine dinucleotide-capped gold nanoclusters: biocompatible photo-emissive nanomaterial and reservoir of lumichrome†

**DOI:** 10.1039/d2na00110a

**Published:** 2022-04-18

**Authors:** Irene Pérez-Herráez, Miguel Justo-Tirado, Mar Bueno-Cuenca, Elena Zaballos-García, Julia Pérez-Prieto

**Affiliations:** Instituto de Ciencia Molecular (ICMol), Universitat de Valencia Calle Catedrático José Beltrán 2 46980 Paterna Valencia Spain julia.perez@uv.es; Department of Organic Chemistry, Universitat de Valencia Av. Vicent Andrés Estellés s/n 46100 Burjassot Valencia Spain

## Abstract

A novel biocompatible nanohybrid consisting of gold nanoclusters (AuNCs) capped with FAD cofactor molecules exhibits distinctive and unique features compared to those of free FAD. The spherical shape of the AuNC provides a large surface to volume ratio, thereby enabling a huge amount of FAD on the AuNC surface while, in basic media and under nitrogen atmosphere, the considerable curvature of its surface enables light-triggered delivery of lumichrome, which is an effective photosensitizer and fluorescent probe.

## Abstract 2

A novel biocompatible nanohybrid consisting of gold nanoclusters (AuNCs) capped with FAD cofactor molecules exhibits distinctive and unique features compared to those of free FAD.

## Graphical abstract

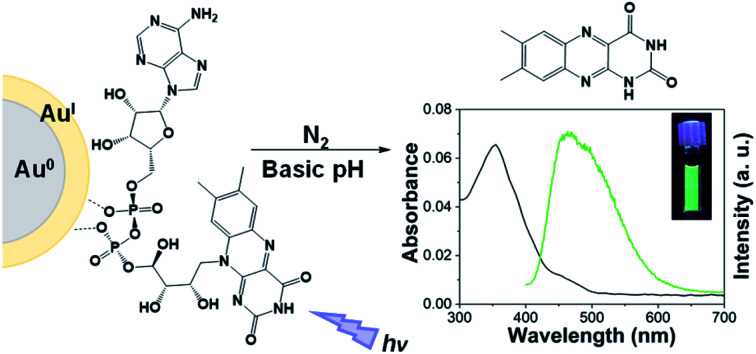

## Introduction

Flavin adenine dinucleotide (FAD) consists of isoalloxazine (Iso) and adenine (Ad) moieties and is a relevant cofactor which catalyses a variety of one- and two-electron redox reactions. This endogenous chromophore exhibits absorption and photoluminescence (PL) spectra which are clearly distinct from those of other cofactors. The Iso ring of FAD determines its strong absorption in the blue and violet wavelength region (at 460 nm and 370 nm, respectively) and its PL (with a broad peak in the 500–600 nm region with the maximum at about 520 nm). In the ground-state (S_0_), the stacked Iso–Ad conformation dominates over the unstacked conformation. Photoisomerization in the singlet excited state (S_1_) converts unstacked molecules to stacked molecules. The two conformations have the same peak wavelengths in absorption and PL. In the pH range from 3 to 10, photo-induced electron transfer occurs from Ad to Iso and as a consequence FAD exhibits a PL quantum yield, *ϕ*_PL_, of about 0.033.^1,2^ The shape of the FAD PL spectrum in aqueous solution and in HeLa cells remains practically unchanged within such a pH range.^1–3^ The PL lifetime of the stacked conformation is several picoseconds while that of the open conformation is of a few nanoseconds.^1,4,5^ Remarkably, FAD has been used in PL lifetime imaging of pH in a single cell, such as a HeLa cell, in which the FAD PL lifetime decreases as the intracellular pH increases;^6^ this can be useful for metabolic investigations since FAD PL permits direct insight into the mitochondrial redox state. In view of this, we envisaged the interest in building FAD-capped nanomaterials in which the occurrence of energy transfer processes between the biomolecule (FAD, see structure in Fig. S1†) and a biocompatible nanomaterial could provide valuable information as well as extending FAD implementation.

**Fig. 1:**
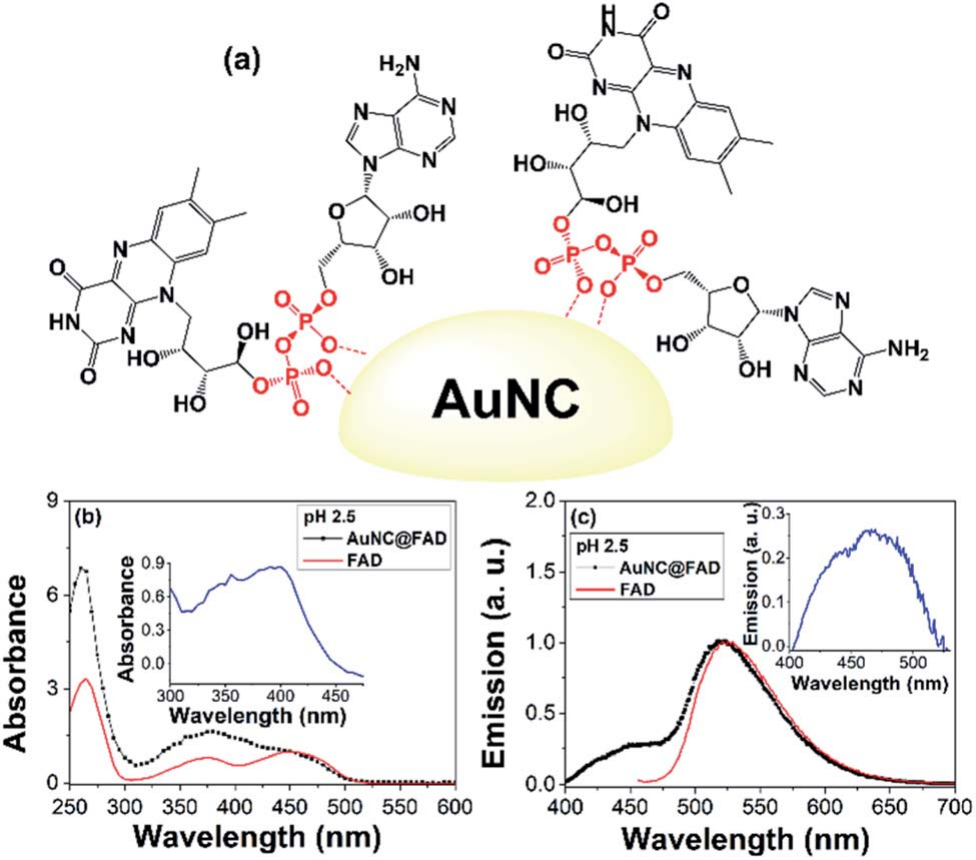
(a) Pictorial representation of the binding of FAD in AuNC@FAD. (b) Absorption and (c) emission spectra (*λ*_exc_ = 390 nm) of AuNC@FAD in water at pH 2.5 (in black) compared to those of FAD in water (in red); insets: difference (b) absorption and (c) emission spectra.

Spherical nanoparticles (NPs) possess a large surface to volume ratio and exhibit a considerable curvature, especially in the case of small NPs. Therefore, a huge amount of FAD could be carried on a NP surface in an otherwise diluted sample (Fig. 1a). In addition, the NP curvature would enable a radial distribution of FAD anchored to the NP surface and it would increase the population of unstacked conformation.

Examples of the use of FAD combined with nanomaterials are nearly non-existent. FAD has been used to immobilize apo-glucose oxidase on the surface of mesoporous NPs *via* specific interactions to control the opening of the NP pores.^7^ In addition, a hybrid nanovector was prepared by Arib *et al.* by encapsulating FAD-Au(iii) complex with a polyethylene glycol (PEG) diacid and then reducing the metal ions to Au(0), thus leading to FAD IN PEG plasmonic gold NPs (AuNPs).^8^ Moreover, a nanomaterial working as a nanolamp (*i.e.*, external fluorophore) has enabled the monitoring of FAD consumption during enzymatic reactions using 980 nm photoexcitation.^9^

Gold nanoclusters (AuNCs) are non-plasmonic NPs of less than 3 nm in diameter and exhibit remarkable properties, such as water solubility, low toxicity, biocompatibility and chemical and photochemical stability. These features make them of great interest for biomedical applications (bioimaging, target-specific treatment, among others).^10^ Their excitation spectrum shows a band in the 280–400 nm range.^11^ Although bare AuNCs show no PL,^12^ organic capping of their surface can convert them into nanofluorophores. The ligand can also play additional roles,^13^ such as to enable targeted delivery of functional molecules^14^ and provide an additional PL peak for ratiometric sensing.^11,15^ Sensitized emission of FAD by AuNC is expected in nanohybrids comprising AuNC and FAD because of the overlap between the AuNC PL and FAD absorption.

Here we focus on the synthesis and (photo)physical characterization of a biocompatible nanofluorophore, namely FAD-capped AuNCs (AuNC@FAD), and demonstrate that under visible light, in relatively basic media and in the absence of oxygen (hypoxia), the nanohybrid efficiently generates lumichrome (LC), which is a suitable photosensitizer and fluorescent probe.

## Results and discussion

### Synthesis and characterization of AuNC@FAD nanohybrid

Briefly, colloidal AuNC@FAD NCs were synthesized *via* a top-down strategy,^11,15^ consisting of (i) the preparation of plasmonic AuNP@FAD NPs by reducing HAuCl_4_ (aqueous solution) with HEPES (10 mM) in the presence of FAD (see ESI for further details†); (ii) separation of the NPs by centrifugation and (iii) treatment of the water colloid with HCl (1 M) leading to etching of the NPs (see Fig. S2†). Centrifugation of the mixture led to a luminescent supernatant with a 2.5 pH, whose optical features were consistent with the formation of AuNC@FAD. Thus, the UV-vis absorption spectrum of these NCs displayed strong bands (at 370 nm and 450 nm). Fig. 1b shows the comparison between the absorption spectrum of FAD and AuNC@FAD dispersions; the different shape of the AuNC@FAD spectrum, with a stronger absorption at 365 nm than that at 450 nm, can be attributed to the contribution of the AuNC absorption (see inset in Fig. 1b showing the difference spectrum between AuNC@FAD and FAD).

The PL spectrum of AuNC@FAD (*λ*_exc_ at 390 nm) exhibited a strong emission at 520 nm, ascribed to FAD (see inset in Fig. 1c), and a shoulder at shorter wavelengths ascribed to the AuNC (average lifetimes, *τ*_av_, of *ca.* 3.9 and 3.0 ns, respectively). The PL quantum yield (*ϕ*_PL_) of AuNC@FAD at *λ*_exc_ = 390 nm was of 0.059, nearly double than that of FAD.

Transmission electron microscopy (TEM) images of AuNC@FAD showed the formation of 2.7 ± 0.9 nm-sized NCs (Fig. S3a†). The Au4f spectrum of the NCs (Fig. S3b†) was consistent with the presence of Au(i) and Au(0), with peaks at 84.58 and 88.18 eV and 83.88 and 87.58 eV, respectively.^12,16,17^ Moreover, the P2p and S2p XPS spectra (Fig. S3b†) showed the presence of P (peak at 134.2 eV)^12^ and S (peaks at 163.18 and 168.58 eV),^18,19^ which can be attributed to FAD and remaining HEPES, respectively.

High resolution TEM (HRTEM) images of the AuNC@FAD showed the crystallinity of the samples and the distance between planes characteristic of AuNCs (Fig. S4, ESI†).

ICP-MS analysis (Table S1†) provided a rough estimation of FAD molecules per AuNC of about 4000,^20^ suggesting a packed organic shell. The formation of this shell could be facilitated by the preference of FAD to exist in water solutions in the folded conformation, in which the aromatic rings are in close proximity.^2,21^ The ^1^H-NMR spectra of AuNC@FAD at different pHs are shown in Fig. S5.† The ^1^H-NMR spectra of FAD, HEPES and the gold salt are shown in Fig. S6–S8.†

### Study of the photostability of the AuNC@FAD nanohybrid

**Fig. 2:**
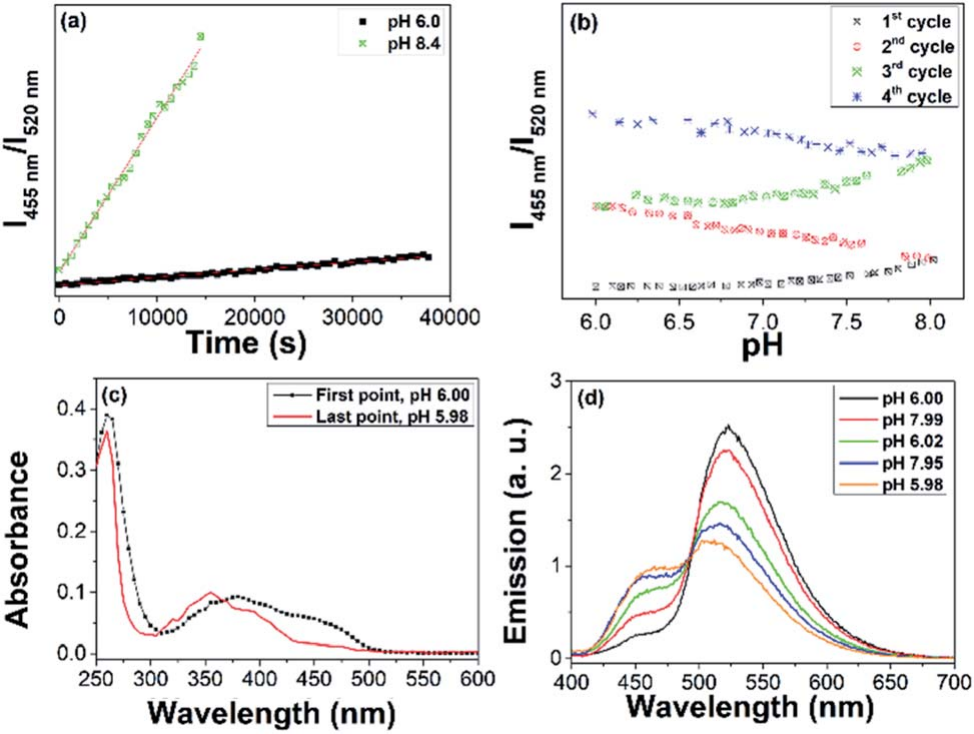
(a) Comparison between the ratiometric response (*I*_455 nm_/*I*_520 nm_) *vs.* time of the AuNC@FAD at pH 6 (in black) over 10 hours and demonstrating the photodegradation of FAD moiety over 4 hours under laboratory light and at pH 8 (in green). (b) Ratiometric response of *I*_455 nm_/*I*_520 nm_*vs.* time of AuNC@FAD for 4 cycles in the 6-to-8 pH range (total of 10 h). (c) Comparison between the absorption spectra of AuNC@FAD and that after 10 h. (d) Evolution of AuNC@FAD emission spectrum (*λ*_exc_ = 390 nm) over the 4 cycles, demonstrating the degradation of FAD and the steadily recovery of the AuNC emission during the 4 cycles.

We studied the photostability of AuNC@FAD considering that flavins, such as FAD, flavin adenine mononucleotide (FMN) and riboflavin (RF) undergo photodegradation in aqueous neutral and in alkaline solutions. The photodegradation quantum yield (*ϕ*_D_) of FAD at pH 8 was of 4.6 ± 0.3 10^−4^, and leads to lumichrome (LC) and lumiflavins (LFs) as the main photoproducts (see structures in the ESI, Fig. S9†). The photostability of AuNC@FAD under laboratory light was monitored for 10 h at pH 6 (Fig. S10†). The comparison between the absorption spectra and between the emission spectra of AuNC@FAD in water at pH 6, before and after irradiation under laboratory light, evidenced (i) the slight decrease of the absorption band in the visible (Fig. S10a†) and (ii) the decrease of the PL at 520 nm combined with a slight increase of that of the AuNC (Fig. S10b†). These results were consistent with slight degradation of FAD moiety to lumiflavin, whose molar absorptivity at 444 nm is lower than that of FAD (10 400 M^−1^ cm^−1^*vs.* 12 530 M^−1^ cm^−1^).^22^ The degradation of the FAD moiety over time was analysed by recording the ratiometric response of *I*_455 nm_/*I*_520 nm_*vs.* time (*k* = 3.08 10^−6^ ± 5.07 10^−8^ s^−1^); see Fig. 2a. Comparatively, FAD showed negligible changes of their optical properties under the same conditions (Fig. S11†).

Subsequently, we analysed the ratiometric response of the emission of AuNC@FAD *vs.* time in the 6–8 pH range for four cycles (from 6-to-8 and 8-to-6, twice over), see Fig. 2b. These studies proved that the degradation of the FAD moiety enhanced in the basic media, leading to LC (Fig. 2c, in red). Because of the transformation of the FAD moiety into LC, the absorption in the visible decreased and the AuNC PL steadily recovered during the 4 cycles (Fig. 2d).

**Fig. 3:**
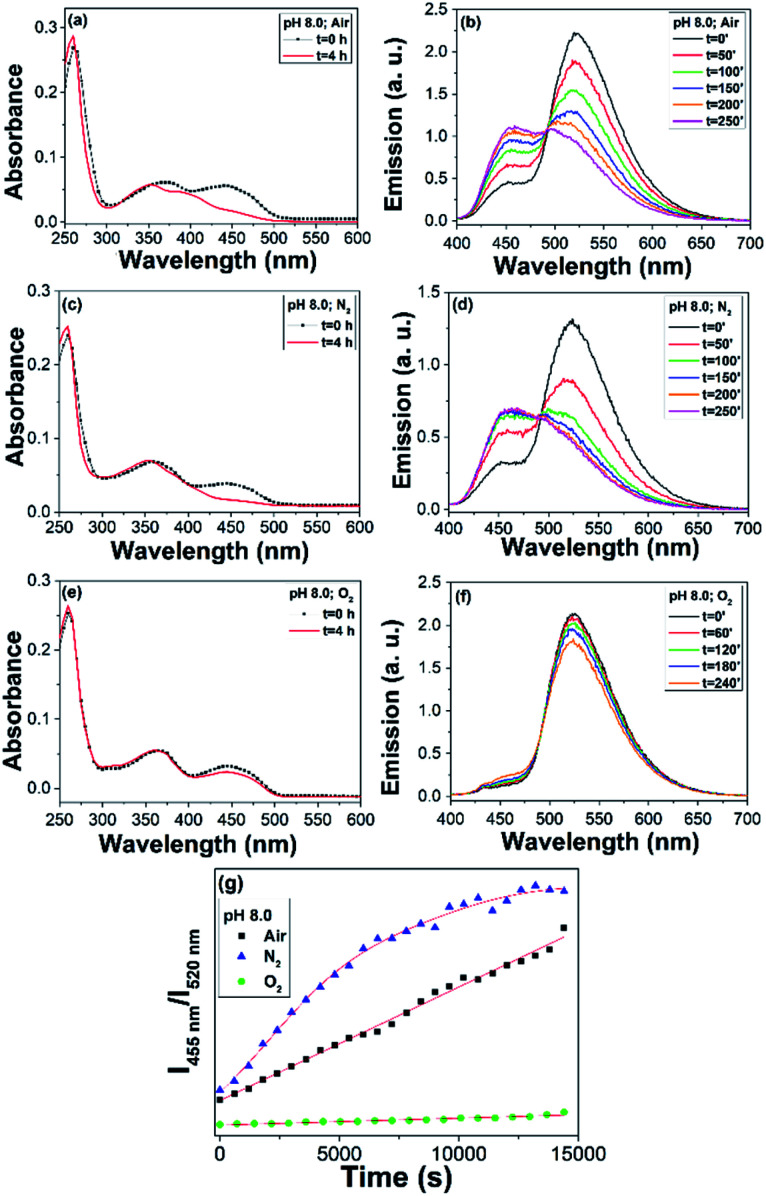
(a) Absorption and (b) emission spectra (*λ*_exc_ = 390 nm) of AuNC@FAD in water and air atmosphere at pH 8 before (in black) and after (in red) irradiation under laboratory light. (c) Absorption and (d) emission spectra (*λ*_exc_ = 390 nm) of AuNC@FAD in water and N_2_ atmosphere at pH 8 before (in black) and after (in red) irradiation under laboratory light. (e) Absorption and (f) emission spectra (*λ*_exc_ = 390 nm) of AuNC@FAD in water and O_2_ atmosphere at pH 8 before (in black) and after (in red) irradiation under laboratory light. (g) Comparison between the ratiometric response (*I*_455 nm_/*I*_520 nm_) *vs.* time of the AuNC@FAD at pH 8 in air (in black), in N_2_ (in blue) and in O_2_ (in green) over 4 hours.

Control studies showed that both FAD and AuNC@FAD at pH 8 were stable under darkness for at least 4 h (Fig. S12 and S13 in ESI†). Moreover, the effect of oxygen concentration on the photodegradation of the FAD moiety was followed by recording the absorption and PL spectra of the nanohybrid at pH 8 for 4 h. Fig. 3a, c and 3e show the absorption spectra of the nanohybrid in water, under air, nitrogen, and oxygen atmosphere, respectively, at pH 8 before (in black) and after (in red) 4 h irradiation under laboratory light, evidencing the role of oxygen concentration in decelerating the degradation of the FAD moiety. Consequently, the evolution of the PL spectra was minimal in oxygen atmosphere. Fig. 3g compares the *I*_455 nm_/*I*_520 nm_ ratiometric response *vs.* time of AuNC@FAD under different oxygen concentrations. The photodegradation of the FAD moiety underwent a 16-fold deceleration of the process in oxygen compared to air (3.90 × 10^−6^ ± 2.58 × 10^−7^ s^−1^*vs.* 6.39 × 10^−5^ ± 1.06 × 10^−6^ s^−1^, respectively), while the process rocketed in nitrogen. Comparatively, in the absence of light, AuNC@FAD exhibited high stability at pH 8 under nitrogen, air, and oxygen atmosphere (Fig. S14†).

It is known that RF photodegradates in water under visible light, but it lacks the Ad unit present in FAD. As a consequence, it leads to the formation of LC and flavins (Fig. S9†) as the photoproducts. RF singlet excited state transforms into LC and carboxymethylflavin *via* intramolecular photodealkylation and intramolecular photoaddition, respectively. RF triplet excited state leads to formylmethylflavin which (i) oxidises to carboxymethylflavin in the presence of oxygen, (ii) transforms in LF under neutral and alkaline media, and (iii) transforms in LC in acid, neutral and alkaline media.^23,24^

FAD exists predominantly in a stacked conformation at pH 8 and the flavin excited state lifetime is of a few ps;^25^ this makes FAD significantly photostable. The enhanced efficiency in the photodegradation of the FAD moiety of the nanohybrid in basic media under nitrogen atmosphere compared to that of FAD could be attributed to a less effective electron transfer process between the Ad and Iso units due to the radial distribution of FAD molecules on the NC surface. In fact, FAD displayed a nearly six-fold lower PL than the FAD moiety on the NC surface at pH 8 (Fig. S15†). It should be noted that LC is an endogenous compound in humans and it is the main photoproduct of RF in neutral and acid media. Because of its effectiveness as a photosensitizer, it has been applied to *in vitro* antibacterial photodynamic therapy.^26^ Lumichrome is an efficient singlet oxygen (^1^O_2_) sensitizer with a ^1^O_2_ quantum yield (*ϕ*_Δ_) of 0.85 in methanol and 0.36 in water at pH 6.^27,28^ In addition, it is a fluorescent probe for fluoride and acetate anions,^29,30^ as well as metal ions.^31^ In short, AuNC@FAD presents distinctive and unique features compared to those of FAD and RF, and its application to biological systems could be of great interest in solid tumours in which hypoxia is a common characteristic.

## Conclusions

A biocompatible nanofluorophore comprising two non-cytotoxic components, namely AuNC and FAD, has been developed to produce a material with distinctive and unique features compared to those of FAD: the spherical shape of the AuNCs plays a crucial role, providing not only a large surface to volume ratio to enable a huge amount of FAD to be carried on the AuNC surface but, especially, a considerable curvature, thus enabling a radial distribution of FAD on the AuNC surface and, as a consequence, increasing the population of unstacked conformations. This resulted in a less efficient electron transfer process between the Iso and Ad moieties of FAD under visible light. In a relatively basic media and in the absence of oxygen (hypoxia), the nanohybrid efficiently delivers LC, which is a suitable photosensitizer and fluorescent probe.

## Author contributions

All authors have given approval to the final version of the manuscript. IPH: investigation, formal analysis; MJT: initial experiments; MBC: initial experiments; EZG: conceptualization, supervision, review & editing; JPP: conceptualization, supervision, writing original draft, funding acquisition.

## Conflicts of interest

There are no conflicts to declare.

## Supplementary Material

NA-004-D2NA00110A-s001

## References

[cit1] Islam S. D. M., Susdorf T., Penzkofer A., Hegemann P. (2003). Chem. Phys..

[cit2] van den Berg P. A. W., Feenstra K. A., Mark A. E., Berendsen H. J. C., Visser A. J. W. G. (2002). J. Phys. Chem. B.

[cit3] Tyagi A., Penzkofer A. (2010). J. Photochem. Photobiol., A.

[cit4] Visser A. J. W. G. (1984). Photochem. Photobiol..

[cit5] Sengupta A., Khade R. V., Hazra P. (2011). J. Photochem. Photobiol., A.

[cit6] Islam M. S., Honma M., Nakabayashi T., Kinjo M., Ohta N. (2013). Int. J. Mol. Sci..

[cit7] Xiao Y., Patolsky F., Katz E., Hainfeld J. F., Willner I. (2003). Science.

[cit8] Arib C., Bouchemal N., Barile M., Paleni D., Djaker N., Dupont N., Spadavecchia J. (2021). Nanoscale Adv..

[cit9] Wilhelm S., del Barrio M., Heiland J., Himmelstof S. F., Galbán J., Wolfbeis O. S., Hirsch T. (2014). ACS Appl. Mater. Interfaces.

[cit10] Kaur N., Aditya R. N., Singh A., Kuo T. R. (2018). Nanoscale Res. Lett..

[cit11] Cuaran-Acosta D., Londoño-Larrea P., Zaballos-García E., Pérez-Prieto J. (2019). Chem. Commun..

[cit12] Londoño-Larrea P., Vanegas J. P., Cuaran-Acosta D., Zaballos-García E., Pérez-Prieto J. (2017). Chem. - Eur. J..

[cit13] Huang Z., Wang M., Guo Z., Wang H., Dong H., Yang W. (2018). ACS Omega.

[cit14] Ding C., Xu Y., Zhao Y., Zhong H., Luo X. (2018). ACS Appl. Mater. Interfaces.

[cit15] Bonanno A., Pérez-Herráez I., Zaballos-García E., Pérez-Prieto J. (2020). Chem. Commun..

[cit16] Casaletto M. P., Longo A., Martorana A., Prestianni A., Venezia A. M. (2006). Surf. Interface Anal..

[cit17] Vanegas J. P., Zaballos-García E., González-Béjar M., Londoño-Larrea P., Pérez-Prieto J. (2016). RSC Adv..

[cit18] Ulman A., Ioffe M., Patolsky F., Haas E., Reuvenov D. (2011). J. Nanobiotechnol..

[cit19] Lichtman D., Craig Jr. J. H., Sailer V., Drinkwine M. (1981). Appl. Surf. Sci..

[cit20] Smith A. M., Johnston K. A., Crawford S. E., Marbella L. E., Millstone J. E. (2017). Analyst.

[cit21] Hahn J., Michel-Beyerle M. E., Rösch N. (1998). J. Mol. Model..

[cit22] Sheraz M. A., Kazi S. H., Ahmed S., Qadeer K., Khan M. F., Ahmad I. (2014). Cent. Eur. J. Chem..

[cit23] Kino K., Kobayashi T., Arima E., Komori R., Kobayashi T., Miyazawa H. (2009). Bioorg. Med. Chem. Lett..

[cit24] Sheraz M. A., Kazi S. H., Ahmed S., Anwar Z., Ahmad I. (2014). Beilstein J. Org. Chem..

[cit25] Li G., Glusac K. D. (2009). J. Phys. Chem. B.

[cit26] Valerón Bergh V. J., Bruzell E., Hegge A. B., Tonnesen H. H. (2015). Pharmazie.

[cit27] Sikorska E., Khmelinskii I. V., Prukala W., Williams S. L., Patel M., Worrall D. R., Bourdelande J. L., Koput J., Sikorski M. (2004). J. Phys. Chem. A.

[cit28] Sikorski M., Sikorska E., Koziolowa A., Gonzalez Moreno R., Bourdelande J. L., Steer R. P., Wilkinson F. (2001). J. Photochem. Photobiol., B.

[cit29] Song P. S., Sun M., Koziolowa A., Koziol J. (1974). J. Am. Chem. Soc..

[cit30] Mizkolczy Z., Biczók L. (2005). Chem. Phys. Lett..

[cit31] Bairi P., Chakraborty P., Roy B., Nandi A. K. (2014). Sens. Actuators, B.

