# Supplementary material for: Flavin adenine dinucleotide-capped gold nanoclusters: biocompatible photo-emissive nanomaterial and reservoir of lumichrome
Source: Nanoscale Adv. 2022 Apr 18;4(10):2250–4. doi: 10.1039/d2na00110a (PMC9418107; doi:10.1039/d2na00110a)
Supplement: NA-004-D2NA00110A-s001 [file NA-004-D2NA00110A-s001.pdf]

Electronic Supplementary Material (ESI) for Nanoscale Advances.
This journal is © The Royal Society of Chemistry 2022

Supporting Information

Flavin Adenine Dinucleotide-capped Gold Nanoclusters: Biocompatible Photo-emissive Nanomaterial and Reservoir of Lumichrome

Irene Pérez-Herráez,[a,b] Miguel Justo-Tirado,[a,b] Mar Bueno-Cuenca,[b] Elena Zaballos-García,*[b] Julia Pérez-Prieto*[a]

[a]Instituto de Ciencia Molecular (ICMol), Universitat de Valencia, Calle Catedrático José Beltrán 2, 46980, Paterna, Valencia, Spain
[b]Department of Organic Chemistry, Universitat de Valencia, Av. Vicent Andrés Estellés s/n, 46100, Burjassot, Valencia, Spain

Page S2. Materials and methods.

Page S3. Structure of FAD (Figure S1). Synthesis of AuNC@FAD (Figure S2).

Page S4. Transmission electron microscopy (TEM) images and X-ray photoelectron spectrum (XPS) of AuNC@FAD (Figure S3).

Page S5. High resolution transmission electron microscopy (HRTEM) images and X-ray dispersive analysis (EDAX) of AuNC@FAD (Figure S4).

Page S6. Content of Au and FAD ligand in the nanoclusters (Table S1).

Page S7. Comparison between the ^{1}H-NMR spectrum at pH 2.5, 6 and 8 of AuNC@FAD (Figure S5).

Page S8. Comparison between the ^{1}H-NMR spectra of HEPES and Au^{3+}+HEPES (Figure S6).

Page S9. Comparison between the ^{1}H-NMR spectra of FAD and Au^{3+}+FAD (Figure S7).

Page S10. Comparison between the ^{1}H-NMR spectra of FAD, HEPES, FAD+HEPES and Au^{3+}+FAD+HEPES (Figure S8).

Page S11. Structure of LC and LFs (Figure S9). Photostability of AuNC@FAD under laboratory light at pH 6 (Figure S10).

Page S12. Photostability of FAD under laboratory light at pH 6 (Figure S11).

Page S13. Stability of FAD under darkness at pH 8 (Figure S12). Stability of AuNC@FAD under darkness at pH 8 (Figure S13).

Page S14. Stability of AuNC@FAD in darkness under different atmospheres at pH 8 (Figure S14).

Page S15. Comparison between AuNC@FAD and FAD emission at pH 8 (Figure S15).

Materials and methods

All chemicals used were of analytical grade. Gold (III) chloride trihydrate ($HAuCl_4 \times 3H_2O$), (2-hydroxyethyl)-1-piperazineethanesulfonic acid (HEPES-H), flavin adenine dinucleotide (FAD), sodium hydroxide (NaOH) and hydrochloric acid (HCl) were purchased from Sigma-Aldrich. All other reagents were of analytical reagent grade and were used as received. In all cases the glassware used in the following procedures was cleaned in a bath of freshly prepared solution of HNO_3-HCl (1:3, v/v) and rinsed thoroughly in water prior to use. For all aqueous solutions, high purity deionized water from Millipore system was used. Nanoparticles and nanoclusters solutions were centrifuged in an Optima Max Ultracentrifuge with MLA-130 Rotor for the time indicated in each case. The supernatant was collected with care to avoid disturbing the red precipitate. The pH measurements were carried out by using a pHmeter (GLP21).

UV/Vis absorption spectra were recorded on a PerkinElmer 1050+ UV/Vis/NIR spectrophotometer. All the data were acquired using 1 cm × 1 cm path length quartz cuvettes. Fluorescence spectra were taken in an Aminco Bowman Series 2 Luminescence spectrophotometer, equipped with a lamp power supply, and working at room temperature. The AB2 software (v.25 5.5) was used to register the data. The quantum yields were measured with a Quantaurus QY Plus c13534-11, equipped with Xenon lamp for excitation in the visible. Time-resolved photoluminescence decays were recorded in a FLS1000 photoluminescence spectrometer (Edinburgh Instruments). The Fluoracle software was used to register the data. All the data of PL decay were acquired using 1 cm × 1 cm path length quartz cuvettes, and specific LED excitation wavelength. Structural and morphological characterizations of AuNC@FAD were performed using bright field transmission electron microscopy (TEM) HT7800 and high-resolution TEM (HRTEM). A field emission gun (FEG) TECNAI G2 F20 microscope, operated at 200 kV, was used. Samples were deposited on carbon films 72 hours prior to measurement in each of the means of dispersion and dried in a vacuum. The diameter of the nanoparticles was determined by ImageJ, in nanometres. Statistical analysis was obtained by measuring the diameter value of 300 nanoparticles. X-ray photoelectron spectroscopy (XPS, K-ALPHA, Thermo Scientific) was used to analyze the samples surface. All spectra were collected using Al-K_ radiation (1486.6 eV), monochromatized by a twin crystal monochromator, yielding a focused X-ray spot (elliptical in shape with a major axis length of 400 µm) at 3 mA × 12 kV. The alpha hemispherical analyzer was operated in the constant energy mode with survey scan pass energies of 200 eV to measure the whole energy band and 50 eV in a narrow scan to selectively measure the particular elements. AuNC@FAD samples were digested in a microwave oven at 200 °C with 100 µL of HNO_3:HCl 1:1 and ultrapure water was added to bring them up to the final volume. The determination of gold, phosphorous and sulfur was performed by ICP-MS in Helio mode, in an Agilent model 7900, using iridium as the internal standard. This test is carried out by the atomic spectroscopy section of the SCSIE of the University of Valencia. ^{1}H-NMR spectra were recorded at 300 or 500 MHz with a Bruker Avance DRX 300 MHz or Bruker Avance DRX 500 MHz spectrometers, using deuterium oxide, 99.9% atom (D_2O) as solvent. The chemical shifts (δ) are reported in ppm.

Structure of FAD

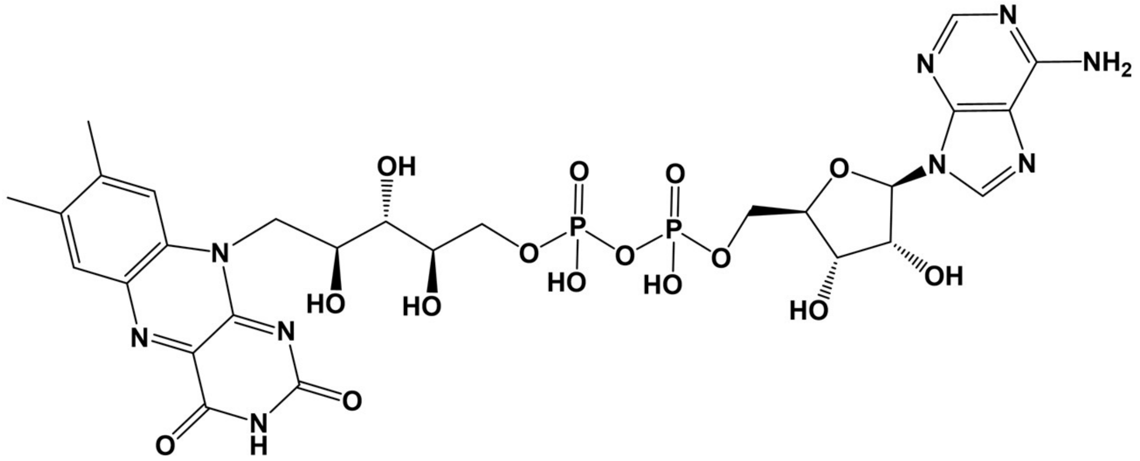

Figure S1. Structure of FAD.

Synthesis of AuNC@FAD

Colloidal AuNCs were prepared by a top-down strategy based on the reduction of Au ions in the presence of reducing agent, in particular HEPES-H and a functional organic ligand, FAD (see below the schematic representation of the process). A 15 ml volumetric falcon flask was filled with reagents in the following order: an aqueous solution of $HAuCl_4$ (50 µl, 50 mM), an aqueous solution of HEPES-H (1 ml, 10 mM) (the solution becomes yellowish) and aqueous solution of FAD (80 µl, 50 mM). The mixture was stirred for 1 min and left to stand for 20 min to obtain a brown aqueous solution. Mixing the aqueous solution with aqueous NaOH (100 µl, 0.5 M) led to red dispersion which contains AuNPs stabilized with FAD (AuNP@FAD). After 24 h resting at room temperature, the red solution was centrifuged at 10000 rpm for 10 min. The red precipitate was resuspended in water (1 ml) and HCl (30 µl, 1 M) was added. The purple solution became rapidly dark grey. After 4 h at room temperature, a yellow supernatant and a black precipitate were observed. The mixture was centrifuged at 5000 rpm for 2 min, separating the precipitate from the yellow supernatant. The supernatant, luminescent under UV light, containing the nanoclusters (AuNC@FAD) was characterized.

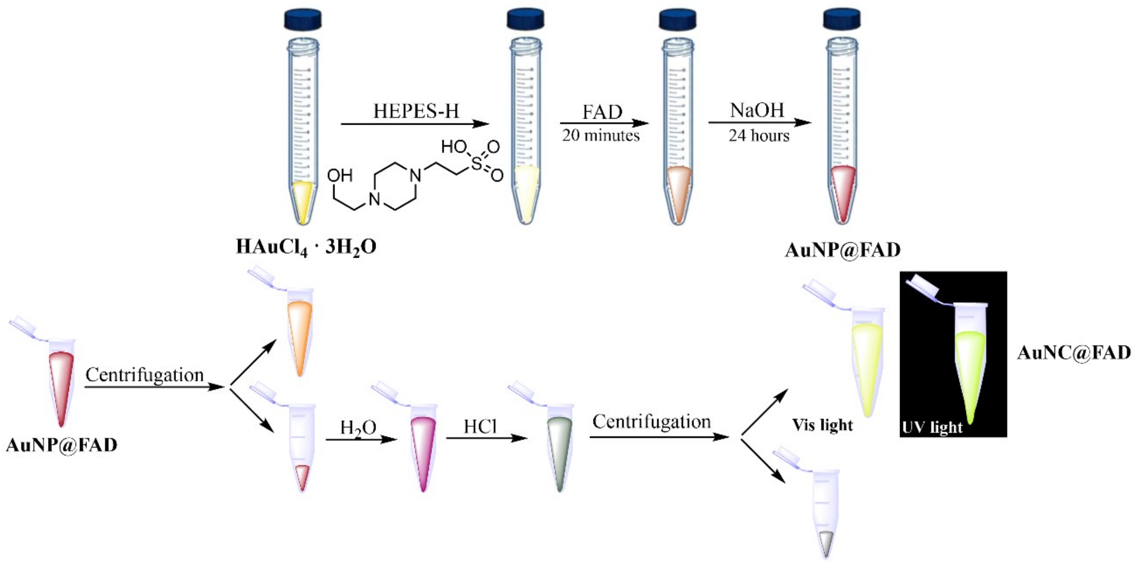

Figure S2. Schematic representation of the process followed for the synthesis of AuNC@FAD.

Transmission electron microscopy (TEM) images and X-ray photoelectron spectrum (XPS) of AuNC@FAD

Figure S3a shows the formation of AuNC@FAD of 2.7±0.9 nm in diameter. The AuNC@FAD sample used to prepare the TEM carbon film was at pH 2.5. Statistical analysis was obtained by measuring the diameter of 300 nanoclusters.

The XPS (Figure S3b) spectra of AuNC@FAD were consistent with the presence of FAD and HEPES on the nanocluster surface.

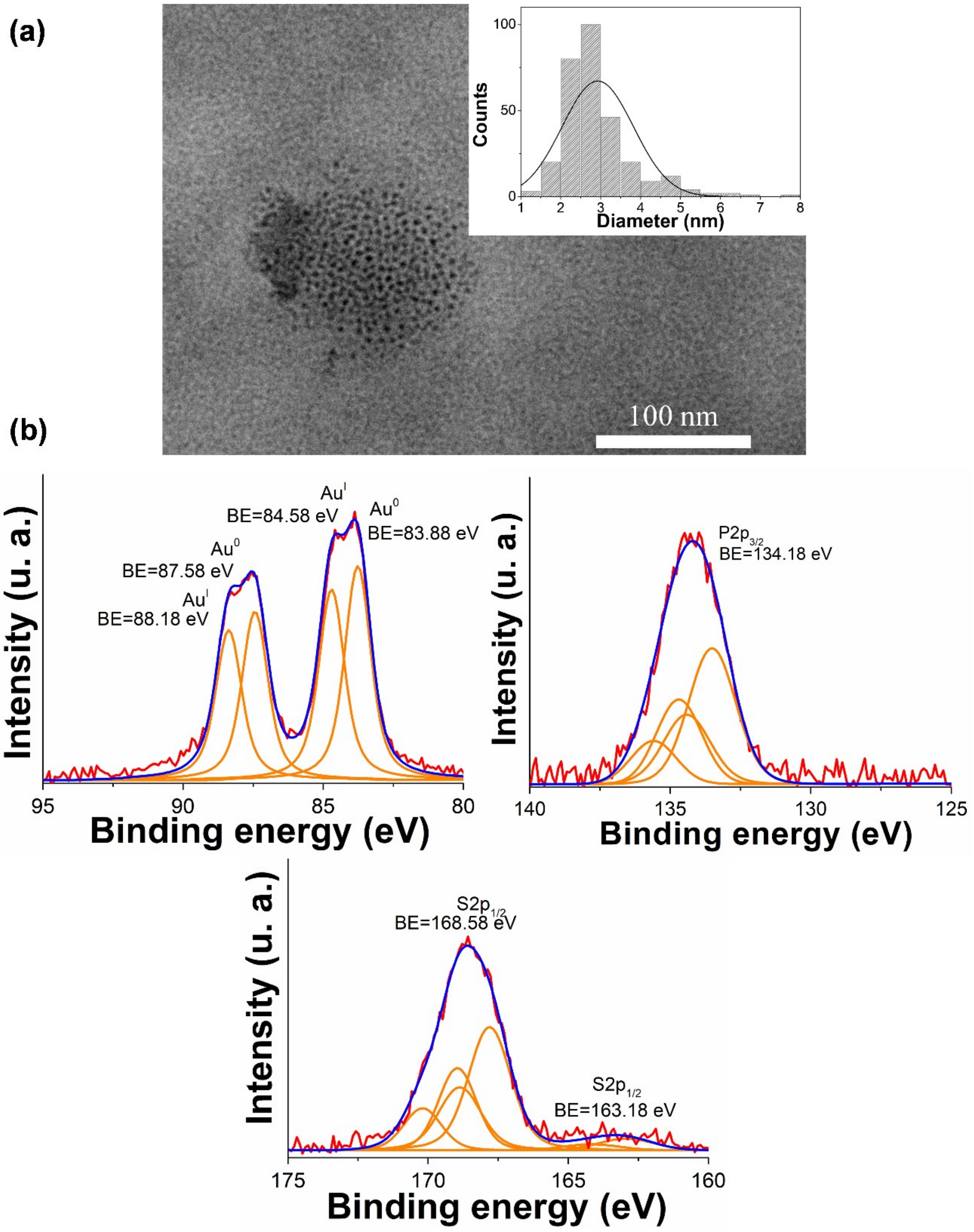

Figure S3. (a) TEM images of AuNC@FAD and histogram with the nanocluster-size distribution. (b) Au4f, P2p and S2p XPS spectra of the AuNC@FAD.

High resolution transmission electron microscopy (HRTEM) images and X-ray dispersive analysis (EDAX) of AuNC@FAD

Figure S4a shows the HRTEM images of AuNC@FAD. The 0.21 nm distance between planes corresponds to the (200) plane of the cubic phase structure of Au. The presence of gold was confirmed by EDAX analysis (Figure S4b).

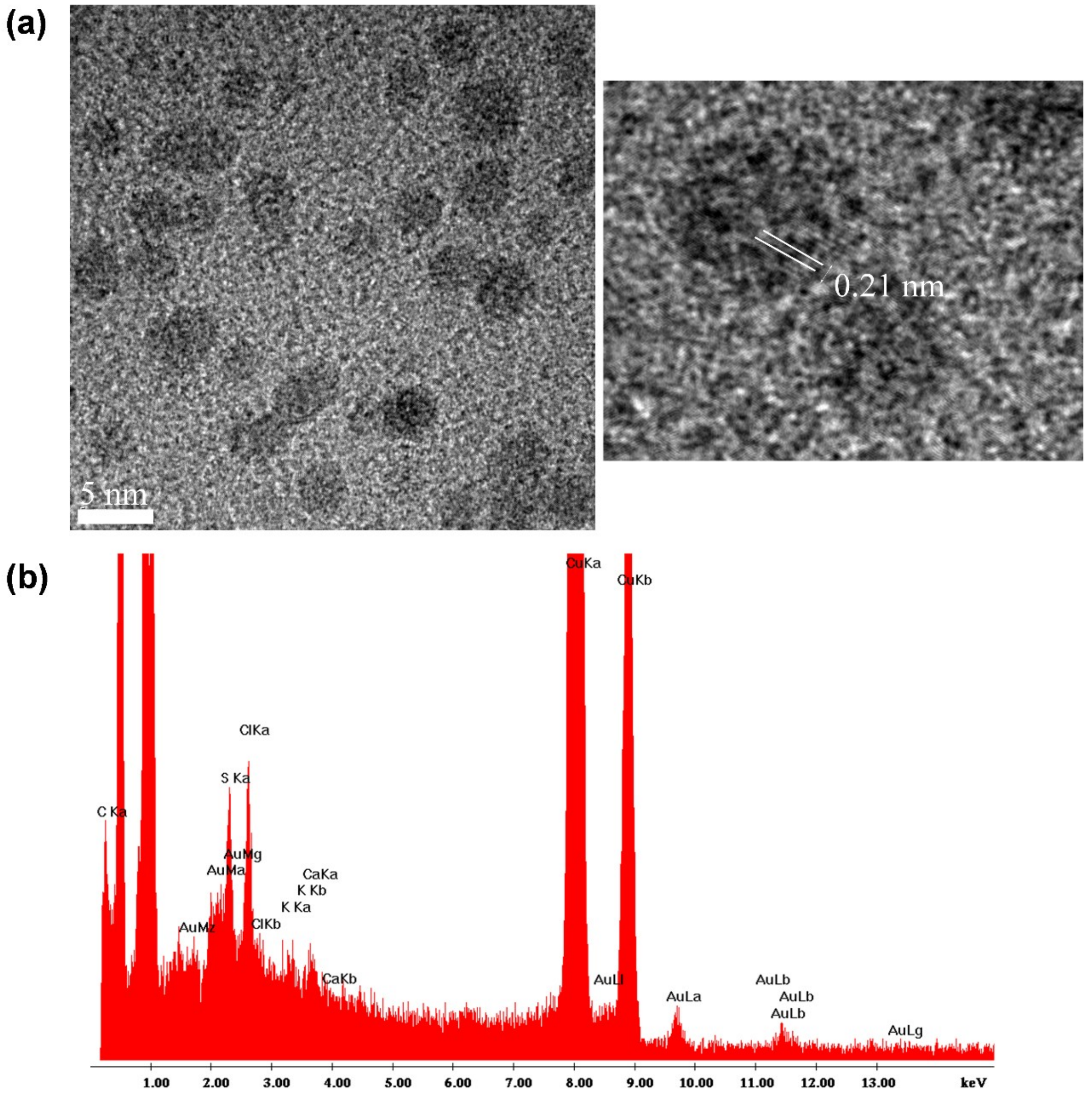

Figure S4. (a) HR-TEM image of AuNC@FAD showing the characteristic distance between planes of Au. (b) EDAX shows the presence of gold in the sample.

Content of Au and FAD ligand in the nanoclusters

Table S1. ICP-MS results.

	Au (µg/L)	P (µg/L)	S (µg/L)
AuNC@FAD	373.4 ± 1.8	791 ± 30	2658 ± 200

- Particle diameter (D)
  $D = 2.7\ nm$
- Particle volume (V)
  $V = \frac{4}{3}\pi(\frac{D}{2})^3 = \frac{4}{3}\pi(\frac{2.7}{2})^3 = 10.20\ nm^3$
- Gold FCC lattice constant (L)
  $L = 0.408\ nm$
- Unit cell volume (U)
  $U = L^3 = (0.408)^3 = 0.06792\ nm^3$
- Number of unit cell (B)
  $B = \frac{V}{U} = \frac{10.30\ nm^3}{0.06792\ nm^3} = 151.649$
- Gold atoms per particle (P)
  $P = 4 \cdot B = 4 \cdot 151.649 = 606.596$
- [Ligand] in sample (H)
  $791 \frac{\mu g}{L} \times 1\ ml \times \frac{1\ L}{1000\ mL} \times \frac{1\ mg}{1000\ \mu g} = 7.91 \times 10^{-4}\ mg\ P$
  $n = \frac{m}{Mr} = \frac{\frac{7.91 \times 10^{-4}\ mg}{1000\ g}}{30.98\ g/mol} = 2.55 \times 10^{-8}\ mol\ P$
  $H = 2.55 \times 10^{-8}\ mol\ P \times \frac{1\ mol\ FAD}{2\ mol\ P} = 1.27 \times 10^{-8}\ mol\ FAD$
- Number of ligands in sample (J)
  $J = H \cdot 6.022 \times 10^{23} = 1.27 \times 10^{-8} \cdot 6.022 \times 10^{23} = 7.688 \times 10^{15}$
- [Au] in sample (A)
  $373.4 \frac{\mu g}{L} \times 1\ mL \times \frac{1\ L}{1000\ mL} \times \frac{1\ mg}{1000\ \mu g} = 3.734 \times 10^{-4}\ mg\ Au$
  $n = \frac{m}{Mr} = \frac{\frac{3.734 \times 10^{-4}}{1000\ g}}{196.97\ g/mol} = 1.896 \times 10^{-9}\ mol\ Au$
  $A = 1.896 \times 10^{-9}\ mol\ Au$
- Number of Au atoms in sample (C)
  $C = A \cdot 6.022 \times 10^{23} = 1.896 \times 10^{-9} \cdot 6.022 \times 10^{23} = 1.142 \times 10^{15}$
- Number of particles in sample (R)
  $R = \frac{C}{P} = \frac{1.142 \times 10^{15}}{606.596} = 1.882 \times 10^{12}$
- Ligands per particle (Y)
  $Y = \frac{J}{R} = \frac{7.688 \times 10^{15}}{1.882 \times 10^{12}} = 4085$

$$4000\ molecules\ FAD/NC$$

Comparison between the ^{1}H-NMR spectrum of AuNC@FAD at pH 2.5, 6 and 8

The ^{1}H-NMR spectrum of AuNC@FAD dispersed in deuterated water proved that the nanohybrid remained stable at pH 2.5 for at least 2 days. The pH of the other samples was adjusted by adding NaOH aliquots.

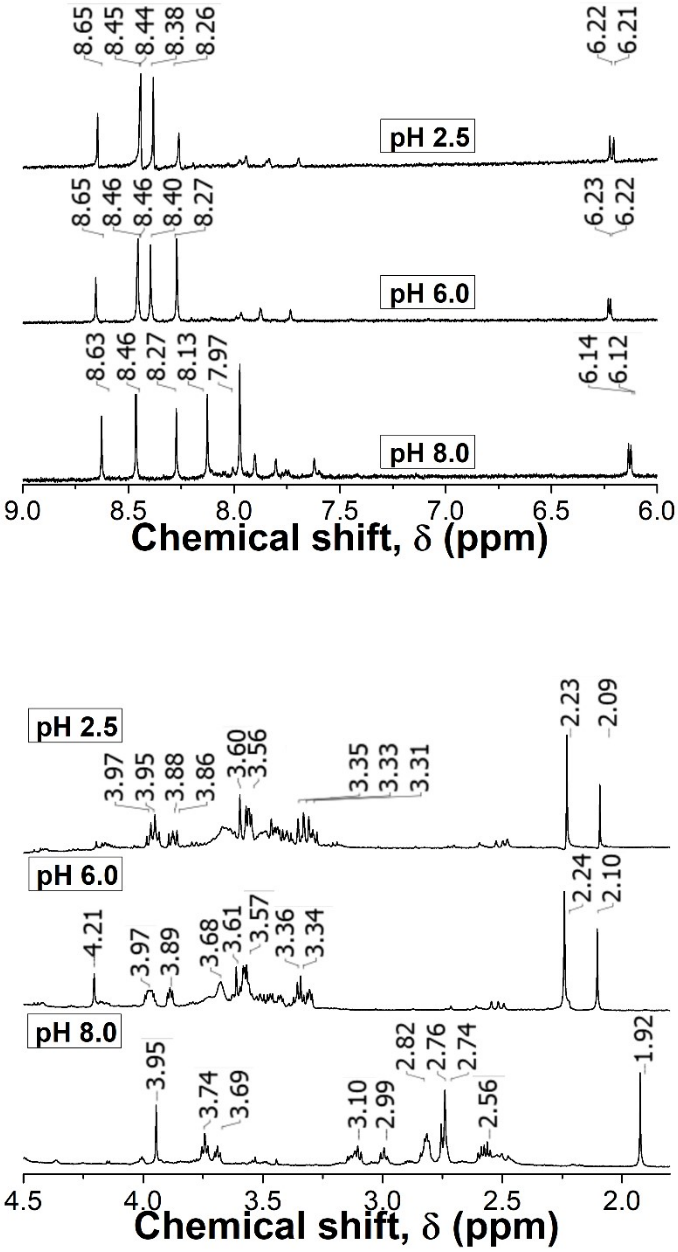

Figure S5. ^{1}H NMR spectra of AuNC@FAD at pH 2.5, pH 6 and pH 8 in the 9.0-6.0 ppm range (top) and in the 4.5-1.8 ppm range (bottom).

Comparison between the ^{1}H-NMR spectra of HEPES and Au^{3+}+HEPES

The ^{1}H-NMR spectrum of HEPES and Au^{3+}+HEPES dispersed in deuterated water showed that mostly the protons 1, 2, 3, 4 and 5 of HEPES shifted to lower field in the presence of Au^{3+}.

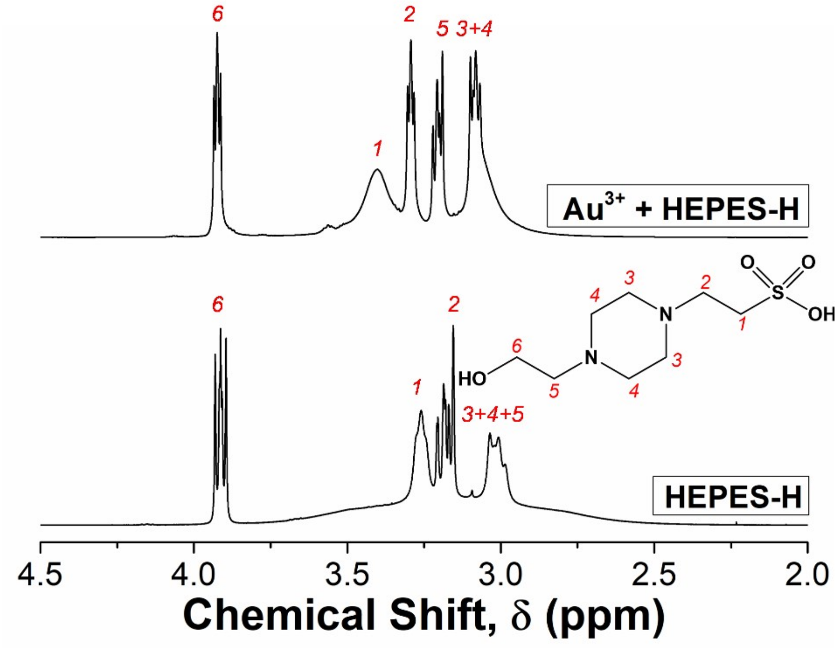

Figure S6. ^{1}H NMR spectra of HEPES and Au^{3+}+HEPES.

Comparison between the ^{1}H-NMR spectra of FAD and Au^{3+}+FAD

The ^{1}H-NMR spectrum of HEPES and Au^{3+}+HEPES dispersed in deuterated water showed that mostly the aromatic protons of FAD shifted to lower field in the presence of Au^{3+}.

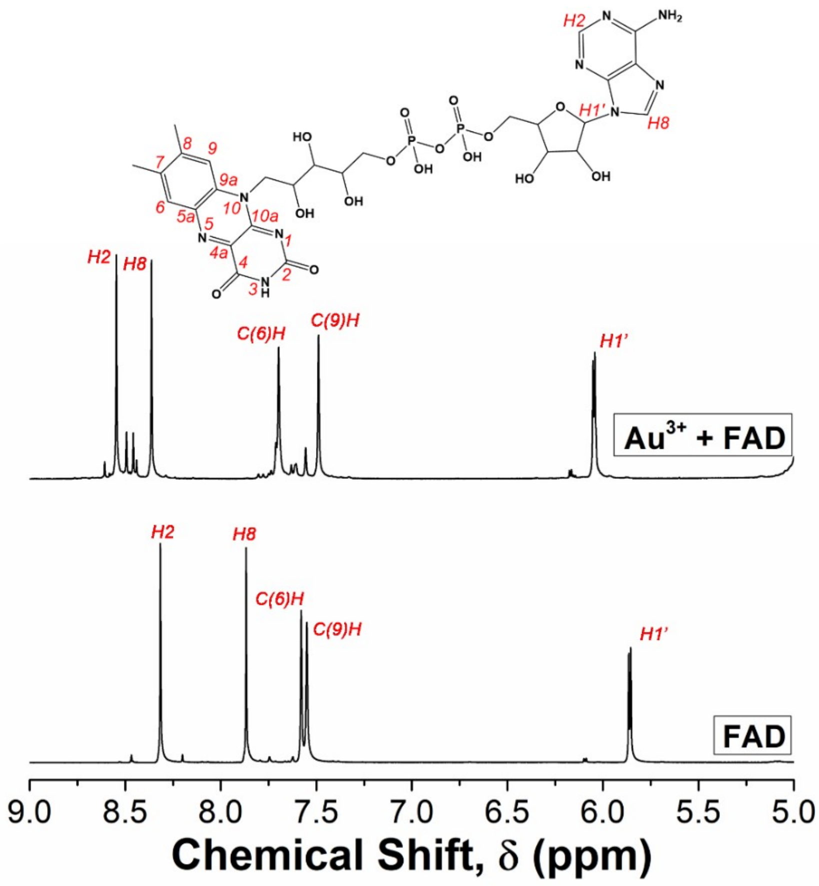

Figure S7. ^{1}H NMR spectra of FAD and Au^{3+}+FAD in the 9.0-5.0 ppm range.

Comparison between the ^{1}H-NMR spectra of FAD, HEPES, FAD+HEPES and Au^{3+}+FAD+HEPES

The ^{1}H-NMR spectrum of HEPES and Au^{3+}+HEPES dispersed in deuterated water showed that FAD protons exhibited negligible shifts in the presence of Au^{3+} and HEPES.

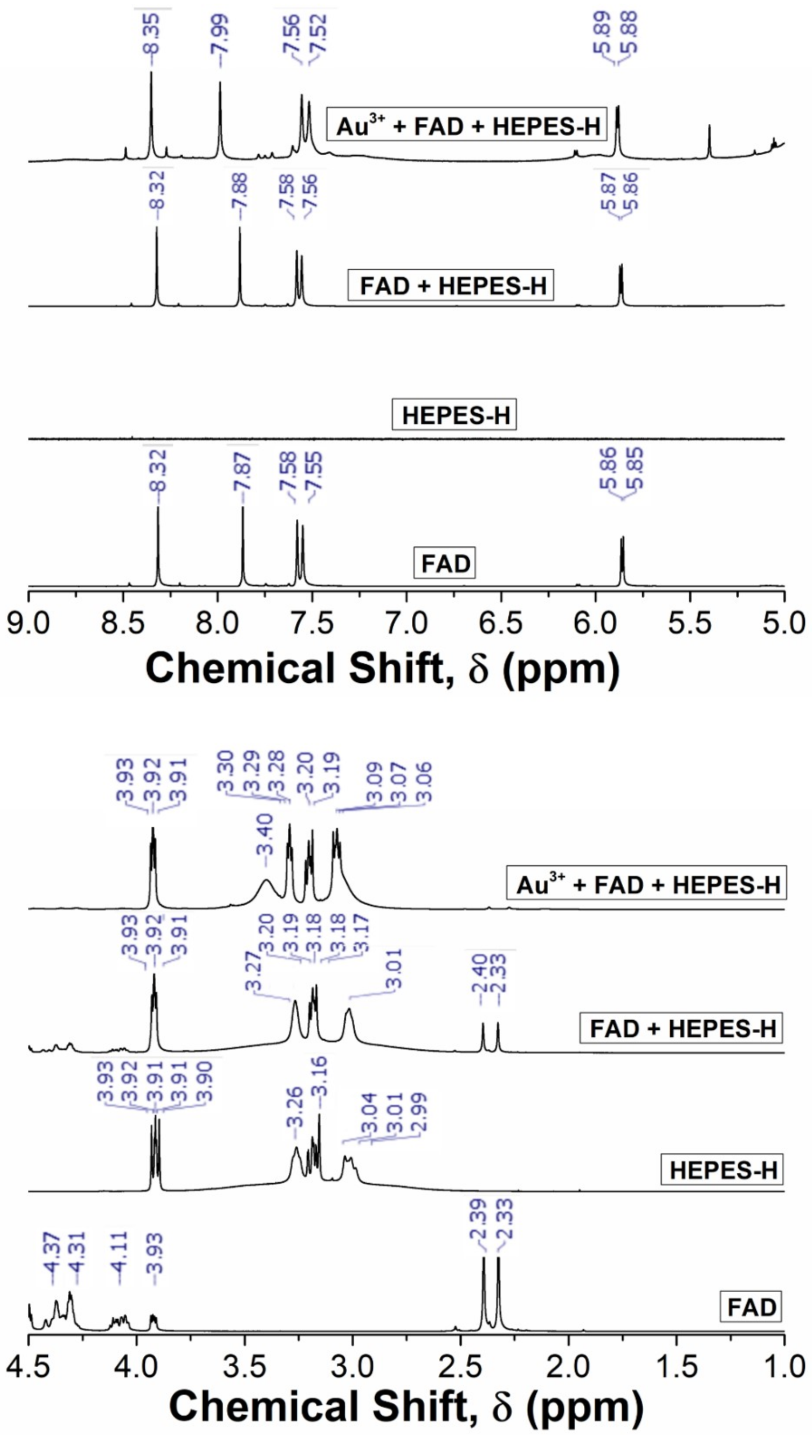

Figure S8. ^{1}H NMR spectra of FAD, HEPES, FAD+HEPES and Au^{3+}+FAD+HEPES in the 9.0-5.0 ppm range (top) and in the 4.5-0.0 ppm range (bottom).

Structure of LC and LFs

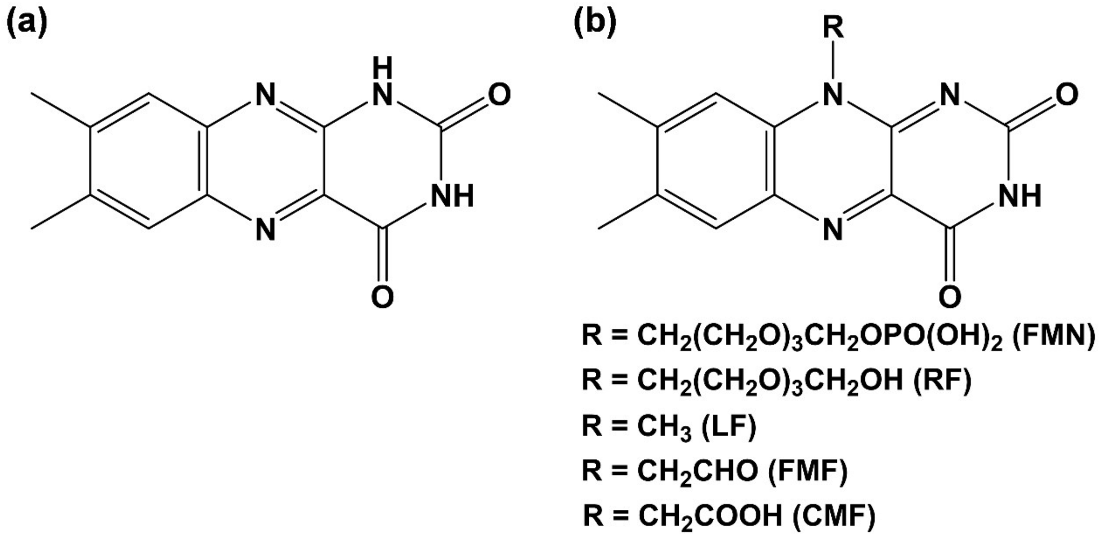

Figure S9. Structure of (a) LC and (b) flavins.

Photostability of AuNC@FAD under laboratory light at pH 6

The absorption and emission spectra of a solution of AuNC@FAD in water at pH 6 were recorded at t = 0 h. Then, the solution was exposed to the laboratory light for 10 hours. The spectra were registered every 10 minutes of light exposure for up to 10 h.

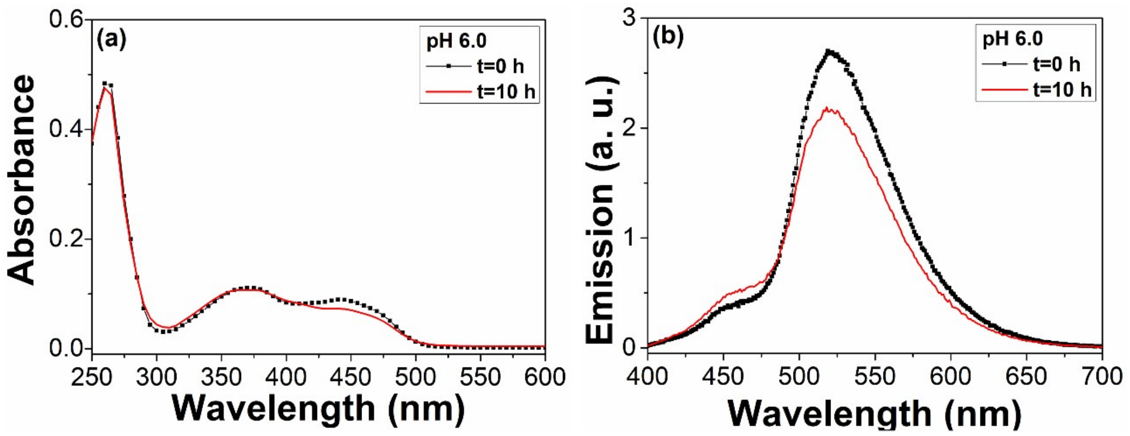

Figure S10. (a) Absorption and (b) emission spectra (λ_{exc} = 390 nm) of AuNC@FAD in water at pH 6 before (in black) and after (in red) exposure to laboratory light.

Photostability of FAD under laboratory light at pH 6

The absorption and emission spectra of a solution of FAD in water at pH 6 were recorded at t = 0 h. Then, the solution was exposed to the laboratory light for 10 hours. The spectra were registered every 10 minutes of light exposure for up to 10 h.

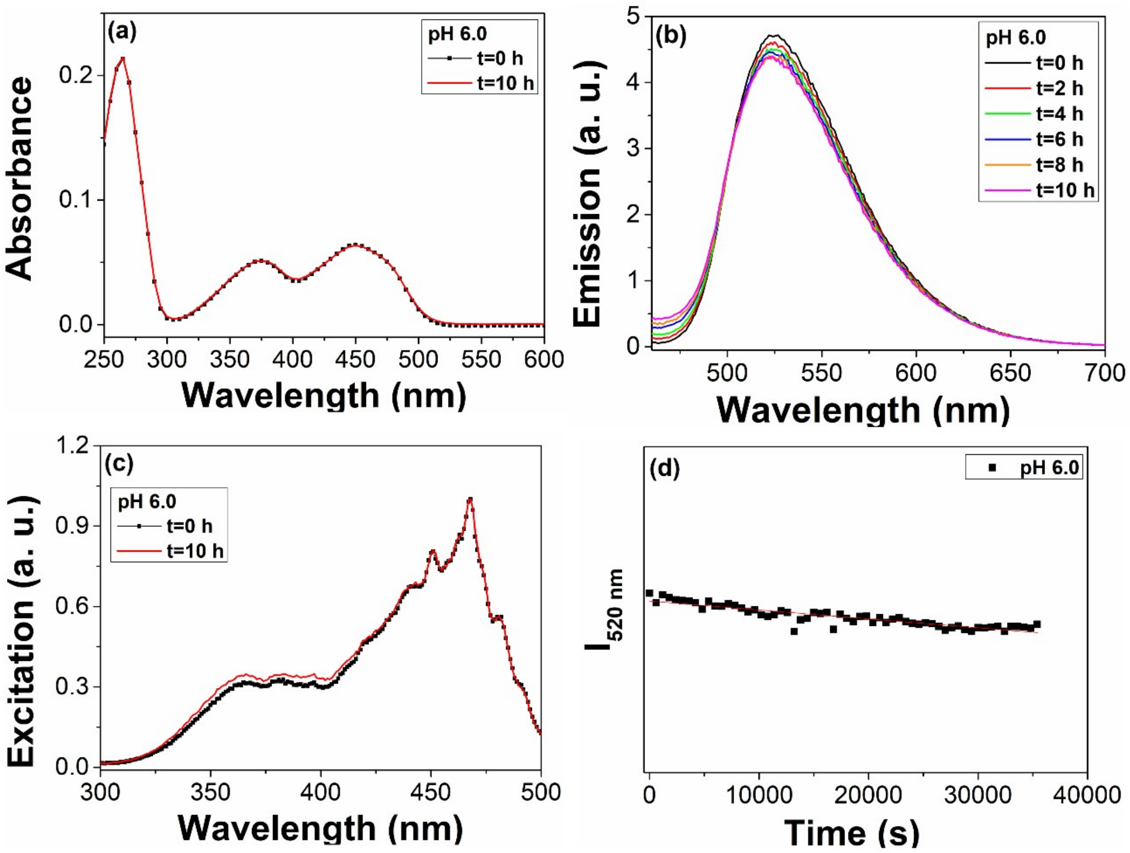

Figure S11. (a) Absorption spectrum of FAD in water at pH 6 before (in black) and after (in red) exposure to laboratory light. (b) Evolution of the emission (λ_{exc} = 390 nm) spectrum of FAD in water at pH 6 under laboratory light. (c) Excitation (λ_{em} = 520 nm) spectra of FAD in water at pH 6 before (in black) and after (in red) exposure to laboratory light. (d) Response ($I_{520\ nm}$) vs. time of the FAD at pH 6 over 10 hours ($k = -8.21E{-}6 \pm 5.17E{-}7\ s^{-1}$).

Stability of FAD under darkness at pH 8

The absorption and emission spectra of a solution of FAD in water at pH 8 were recorded at t = 0 h. The solution was kept in darkness for 4 hours and then the spectra were recorded again.

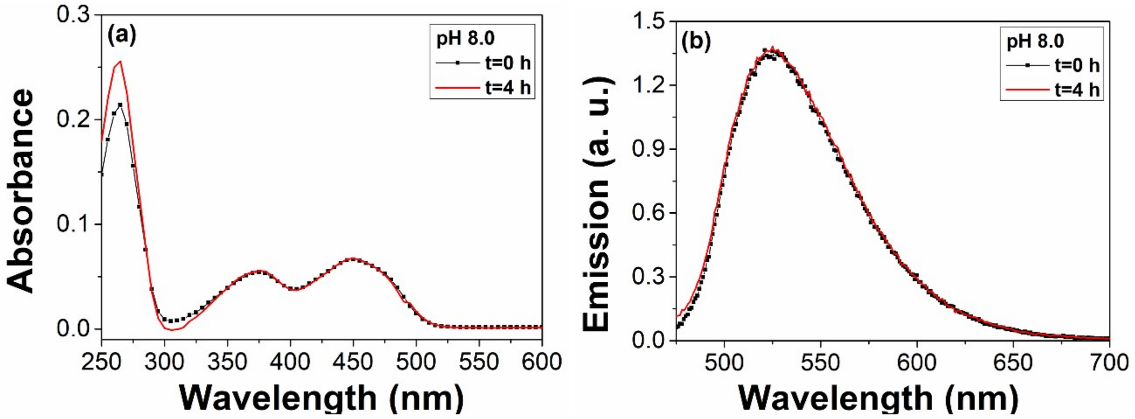

Figure S12. (a) Absorption and (b) emission spectra (λ_{exc} = 390 nm) of FAD in water at pH 8 before and after 4 hours in darkness.

Stability of AuNC@FAD under darkness at pH 8

The absorption and emission spectra of a solution of AuNC@FAD in water at pH 8 were recorded at t = 0 h. The solution was kept in darkness for 4 hours and then the spectra were registered again.

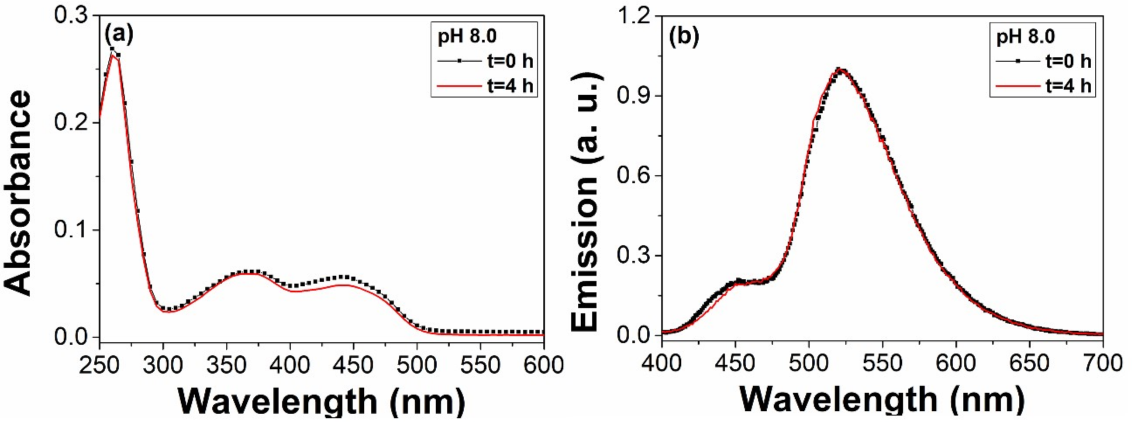

Figure S13. (a) Absorption and (b) emission spectra (λ_{exc} = 390 nm) of AuNC@FAD in water at pH 8 before and after 4 hours in darkness.

Stability of AuNC@FAD in darkness under different atmospheres at pH 8

The absorption and emission spectra of a solution of AuNC@FAD in water at pH 8 under air, N_2 and O_2 atmosphere were recorded at t = 0 h. Then, the solution was kept in darkness for 4 hours and the spectra were recorded again.

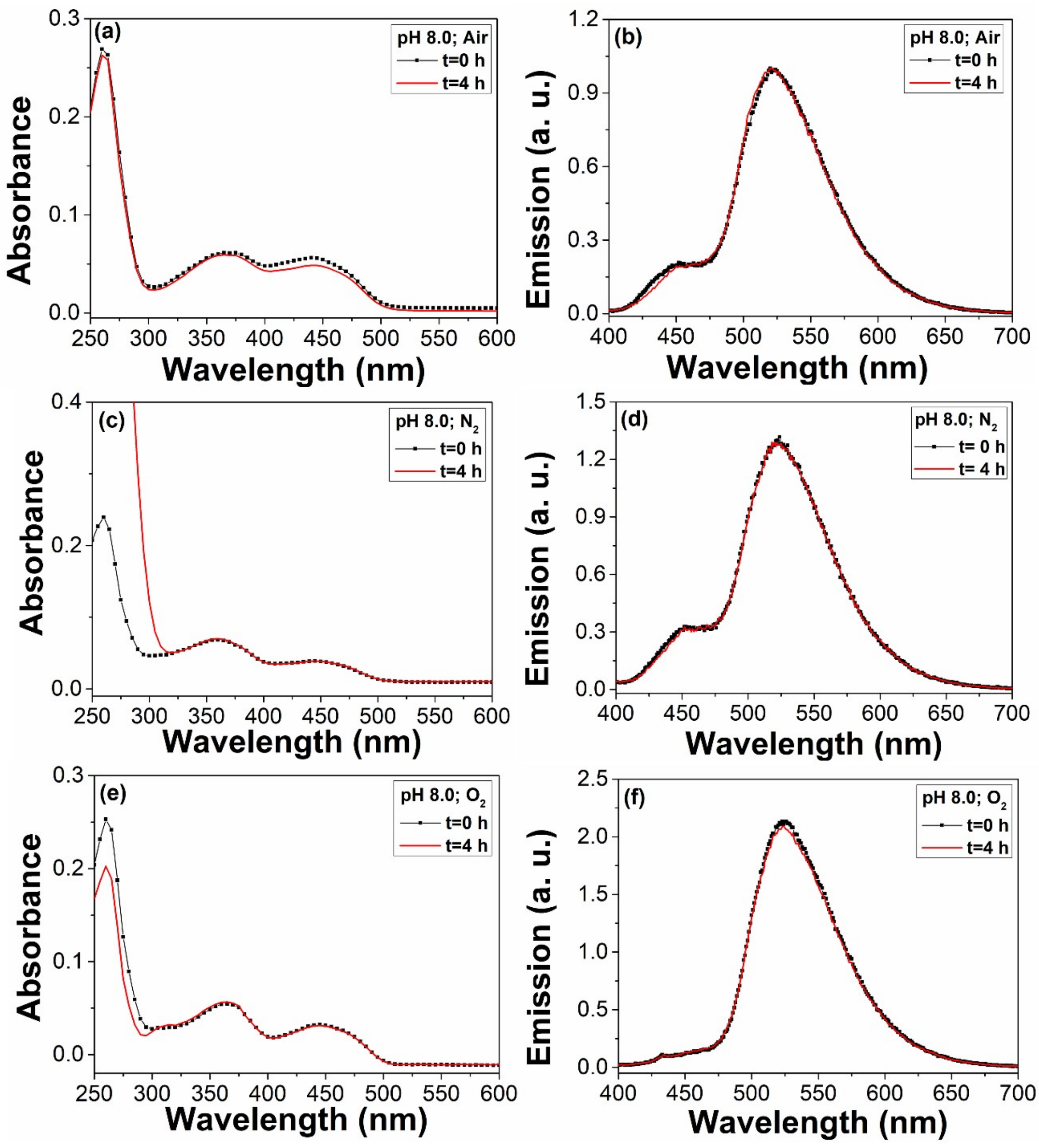

Figure S14. (a) Absorption and (b) emission spectra (λ_{exc} = 390 nm) of AuNC@FAD in water and air atmosphere at pH 8 before and after 4 hours in darkness. (c) Absorption and (d) emission spectra (λ_{exc} = 390 nm) of AuNC@FAD in water under N_2 atmosphere at pH 8 before and after 4 hours in darkness. (e) Absorption and (f) emission spectra (λ_{exc} = 390 nm) of AuNC@FAD in water under O_2 atmosphere at pH 8 before and after 4 hours in darkness.

Comparison between AuNC@FAD and FAD emission at pH 8

The photoluminescence spectra (λ_{exc} = 455 nm) of AuNC@FAD and FAD dispersed in water at pH 8 (absorbance of 0.1 at 455 nm) were registered for comparative purposes.

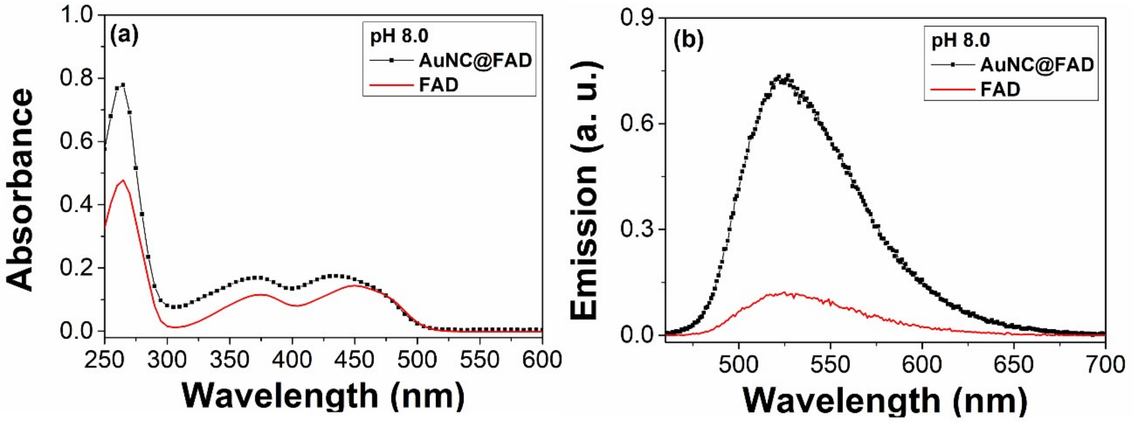

Figure S15. (a) Absorption and (b) emission spectra (λ_{exc} = 455 nm) of AuNC@FAD and FAD in water at pH 8.
